# A New Eucalyptol-Rich Lavender (*Lavandula stoechas* L.) Essential Oil: Emerging Potential for Therapy against Inflammation and Cancer

**DOI:** 10.3390/molecules25163671

**Published:** 2020-08-12

**Authors:** Mohamed Nadjib Boukhatem, Thangirala Sudha, Noureldien H.E. Darwish, Henni Chader, Asma Belkadi, Mehdi Rajabi, Aicha Houche, Fatma Benkebailli, Faiza Oudjida, Shaker A. Mousa

**Affiliations:** 1 The Pharmaceutical Research Institute, Albany College of Pharmacy and Health Sciences, Rensselaer, New York, NY 12144, USA; sudha.thangirala@acphs.edu (T.S.); nour_darwish83@yahoo.com (N.H.E.D.); m.rajabi.s@gmail.com (M.R.); shaker.mousa@acphs.edu (S.A.M.); 2 Département de Biologie et Physiologie Celulaire, Faculté des Sciences de la Nature et de la Vie, Université-Saad Dahlab-Blida 1, Blida 09000, Algeria; hoch.snv@gmail.com (A.H.); benkpharma@gmail.com (F.B.); 3 Hematology Unit, Clinical Pathology Department, Mansoura Faculty of Medicine, Mansoura University, Mansoura 35516, Egypt; 4 Laboratoire de Pharmaco-Toxicologie, Laboratoire National de Contrôle des Produits Pharmaceutiques (LNCPP), Dely-Ibrahim, Algiers 16047, Algeria; hennichader@hotmail.fr; 5 Faculté de Médecine-Université Ben Youcef Ben Khedda-Alger I, Algiers 16000, Algeria; 6 Laboratoire Pharmaco-Toxicologie, Centre de Recherche & Développement Saidal, Algiers 16004, Algeria; santepharmacrd@gmail.com; 7 Laboratoire d’Anatomie Pathologique, Centre Hospitalo-Universitiare de Beni-Messous, Algiers 16206, Algeria; medchem2020@gmail.com

**Keywords:** *Lavandula stoechas* essential oil, topical anti-inflammatory effect, anticancer activity, melanoma cell lines, 1,8-Cineole

## Abstract

Background/Aim: natural products are a potential source for drug discovery and development of cancer chemoprevention. Considering that drugs currently available for the treatment of inflammatory and cancer conditions show undesirable side effects, this research was designed to evaluate, for the first time, the in vitro anticancer activity of Algerian *Lavandula stoechas* essential oil (LSEO) against different cancer cell lines, as well as its in vitro and in vivo topical and acute anti-inflammatory properties. Materials and Methods: the LSEO was extracted by steam distillation, and chemical composition analysis was performed using gas chromatography. The main compounds identified in LSEO were oxygenated monoterpenes, such as 1,8-Cineole (61.36%). LSEO exhibited a potent anti-inflammatory activity using the xylene-induced mouse ear edema model. Results: LSEO (200 and 20 mg/kg) was able to significantly reduce (*p* < 0.05) the carrageenan-induced paw edema with a similar effect to that observed for the positive control. Topical application of LSEO at doses of 82 and 410 mg/kg significantly reduced acute ear edema in 51.4% and 80.1% of the mice, respectively. Histological analysis confirmed that LSEO inhibited the skin inflammatory response. Moreover, LSEO was tested for its antitumor activity against different cancer cell lines. LSEO was found to be significantly active against human gastric adenocarcinoma (AGS), Melanoma MV3, and breast carcinoma MDA-MB-231 cells, with median inhibitory concentration (IC_50_) values of 0.035 ± 0.018, 0.06 ± 0.022 and 0.259 ± 0.089 µL/mL, respectively. Altogether, these results open a new field of investigation into the characterization of the molecules involved in anti-proliferative processes. Conclusion: We suggest that LSEO, with 1,8-Cineole as the major active component, is a promising candidate for use in skin care products with anti-inflammatory and anticancer properties. The results of this study may provide an experimental basis for further systematic research, rational development, and clinical utilization of lavender resources.

## 1. Introduction

Inflammation is regarded as an important baseline reaction responsible for manifestations of various chronic diseases such as cancer, septic shock, diabetes, atherosclerosis, and obesity. Tissue damage determines the development of inflammation by mechanisms that include the production of chemical mediators, the recruitment of specific cells and an increased rate of cell division. These inflammatory mediators, when present in excess, inhibit apoptosis [1] and lead to the loss of tissue homeostasis, which favors the onset of mutations that could lead to cancer development [1,2,3,4].

Recent findings have expanded the concept that inflammation is a serious component of cancer growth and progression. Chronic inflammation has been associated to several steps involved in carcinogenesis, comprising cellular alteration, promotion, proliferation, invasion, angiogenesis, and metastasis [3]. Many cancers arise from sites of infection, chronic irritation, and inflammation. It is now becoming clear that the tumor microenvironment, which is largely orchestrated by inflammatory cells, is an indispensable participant in the neoplastic process, fostering proliferation, survival, and migration [3]. In some types of cancer, the inflammatory process is present before a malignant change occurs; however, in other types of cancer, an oncogenic change induces an inflammatory micro-environment that promotes the development of tumors [1,2].

In this context, drug discoveries of new agents with anti-inflammatory and anticancer properties have a unique interest for medical care. Several in vivo and in vitro models of inflammation and cancer have been used for the discovery of new therapeutic agents. The identification of antitumor or anticancer properties could test the drug in different cancer cell lines with a principal objective of separating features associated with cytotoxic effect toward many cell lines from those that affect only a specific cell type [5,6,7].

Many traditional medicines, phytochemical extracts, essential oils (EOs), and volatile constituents extracted from aromatic herbs and medicinal plants have been widely used as anti-inflammatory, antitumor, antioxidant, and antimicrobial agents for the prevention and treatment of different human diseases [2,5,8]. Several studies have demonstrated the anti-inflammatory and anticancer activities of products derived from plants, such as EOs. Many cytotoxic molecules that are of plant origin are widely used in chemotherapy [2,9]. EOs from some Lamiaceae species, such as lavender, have shown effectiveness in these processes [5,10].

Algeria is a country with many unknown plants whose compounds could be used in medicine [8]. Among the various plants with putative pharmacological properties, lavender species are common in Algeria. The genus *Lavandula* consists of approximately 20 species with more than 100 varieties of lavender. *Lavandula stoechas*, locally known as “El Halhal”, is an evergreen shrub, and it usually grows up to one meter high with spike violet flowers. *L. stoechas*, or wild lavender, is one of the plants with aromatic leaves and attractive bracts at the top of the flowers. It grows in western Mediterranean countries, Algeria, Tunisia, Italy, France, Spain, Turkey, and India [8,11,12,13].

*Lavandula* is an important genus of the Lamiaceae family that comprises EO-producing plants relevant to the food, cosmetic, perfumery, and pharmaceutical industries. *Lavandula stoechas* essential oils (LSEO) from lavender plants have been used for the first aid cure of wounds, abscesses, and burns [11,13]. Recently, Rahmati et al. [14] and Rafiee et al. [15] demonstrated anxiolytic, sedative, and antispasmodic activities. The chemical composition and antimicrobial evaluation of LSEO have been the subject of several studies over the years [12,16,17]. However, there are very few detailed publications on its anti-inflammatory and anticancer properties. To our knowledge, the EO of *L. stoechas* grown in the Cherchell region (North-Center of Algeria) has not yet been reported in the literature. Taking this into account, the present research was designed to evaluate, for the first time, the in vitro anticancer activity of Algerian LSEO against different cancer cell lines as well as its in vitro and in vivo topical and acute anti-inflammatory properties.

## 2. Results and Discussion

### 2.1. Chemical Composition of Lavandula stoechas Essential Oil

**Figure 1:**
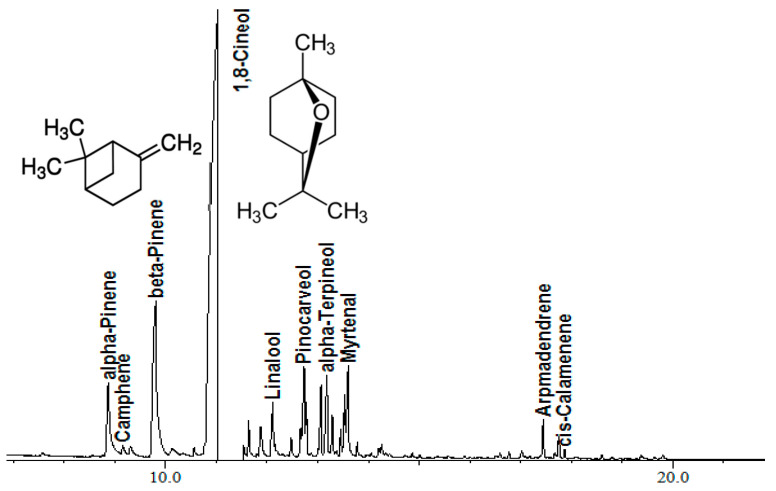
The chemical profile of *Lavandula stoechas* essential oil extracted using steam distillation. (*X*-axis in minutes).

We used EO extracted from the aerial parts of *Lavandula stoechas*. Determination of the chemical composition of LSEO was done using gas chromatography-mass spectrometry (GC-MS), and quantitative and qualitative compositions are shown in Table 1 and Figure 1.

Different constituents were detected and quantified, and 21 compounds were identified. The LSEO consisted mainly of oxygenated monoterpenes (79.23%) and low amounts of hydrocarbons (1.84%). Eucalyptol (1,8-cineole) was found to be the major component (61.36%), followed by β-pinene (13.83%) and α-pinene (4.75%). Other compounds were detected but were less than 3% (Table 1). Thus, LSEO from Algeria extracted by steam distillation may be classified as a “Eucalyptol Chemotype”.

*L. stoechas* has been the object of several phytochemical studies that have pointed out a high chemical variability, allowing the establishment of several chemotypes. LSEO is characterized by significant variations in the amounts of fenchone, camphor, and 1,8-cineole, and the fenchone/camphor chemotype is the most commonly identified [8,17,18].

Our findings are in discordance with others carried out on *Lavandula stoechas* collected from other regions worldwide [16,17,19]. Indeed, others studies reported the richness of LSEO in fenchone and camphor as the major constituents. Vokou et al. [16] reported that *L. stoechas* is rich in EO, and the principal compounds were fenchone (41%), 1,8-cineole (29%), and α-pinene (1.6%). Ristorcelli et al. [20] reported the chemical composition of the EOs from 50 samples of *L. stoechas* from different areas of Corsica (France) during the flowering stage; they found important variations in the major constituents:  fenchone, 15–75%; camphor, 2–56%; and 1,8-cineole, 1–8%. The variations detected in LSEO between our research and others are surely related to the disparity in the area of collection. This may be explained by the influence of the external environment on the synthesis and regulation of secondary metabolism pathways in medicinal plants. In fact, the chemical composition of medicinal flora differs with phenological transformations, harvestings area, collected parts, and methods of distillation [13,19].

### 2.2. Anti-Inflammatory Activity In Vitro

#### 2.2.1. Irritation Test in Red Blood Cell System Cellular Model

The results of in vitro anti-inflammatory activity determined by the human red blood cell membrane stabilization method were shown in Table 2. The LSEO showed a concentration dependent anti-inflammatory activity, and the protection percent increased with an increase in the concentration of the samples. At a concentration of 3 µL/mL, the LSEO produced 74.471 ± 0.465% inhibition of human red blood cells (HRBC) hemolysis (*p* < 0.05) as compared with 27.552 ± 3.354% produced by standard NSAID sodium diclofenac. However, when comparing IC_50_ values, it is clear from the data that sodium diclofenac showed greater response than LSEO.

The LSEO exhibited a membrane stabilization effect by inhibiting hypotonicity-induced lysis of HRBC membrane. This membrane is analogous to the lysosomal membrane and its stabilization implies that the LSEO may stabilize lysosomal membranes as well. Stabilization of lysosomal membrane is essential in decreasing the inflammatory reaction by stopping the discharge of lysosomal components of activated neutrophil such as bactericidal enzymes and proteases, which cause further tissue inflammation and destruction upon extracellular release [21,22]. Although the exact mechanism of the membrane stabilization by the LSEO extract is not known yet, hypotonicity-induced hemolysis may arise from shrinkage of the cells due to osmotic loss of intracellular electrolyte and fluid components. The LSEO may inhibit the processes, which may stimulate or enhance the efflux of these intracellular components [23]. Karthik et al. [24] have reported that the lavender EO presented RBC membrane stabilization action by preventing hypotonicity-induced lysis of erythrocyte membrane. Both the lavender EO and the positive control (NSAID) displayed anti-inflammatory activity, but the NSAID being more effective. Erythrocyte membrane stabilization is an important mechanism to inhibit the leakage of cellular constituents (protein and fluids) into the tissues during a time of increased penetrability initiated by inflammatory mediators.

#### 2.2.2. Inhibition of Denaturation of Bovine Serum Albumin

Denaturation of protein is a recognized source of inflammation. Therefore, as part of the examination to assess the anti-inflammatory mechanism of LSEO, its aptitude to inhibit BSA denaturation was calculated. The inhibitory action of different concentration of LSEO on BSA denaturation is shown in Table 3. It has been found that denaturation of BSA is inhibited by several NSAIDs such as indomethacin and salicylic acid, proving this assay to be useful in the detection of other anti-inflammatory compounds. It was detected from this assay that eucalyptol rich fraction of LSEO presented a dose-dependent maximum inhibition of denaturation of BSA of 72.625 ± 2.56% at 0.4 µL/mL and a standard NSAID (sodium diclofenac) revealed maximum inhibition of 76.117 ± 0.534% at the 0.01 mg/mL. On the basis of these results, LSEO showed significant anti-inflammatory activity (IC_50_ = 2.447 ± 0.873 µL/mL, *p* < 0.05) as compared to NSAID control (IC_50_ = 8.260 ± 0.943 µL/mL), and suggests that LSEO has potential anti-inflammatory activity.

The most commonly used drugs for management of inflammatory conditions are NSAIDs and steroids, which have several adverse effects, especially gastric irritation leading to formation of gastric ulcers [21,22,23]. Denaturation of tissue proteins is one of the well-documented causes of inflammation. The preliminary screening with the BSA assay indicated that LSEO has significant levels of protection against heat denaturation of the protein. Therefore, protection against protein denaturation, which was the central mechanism of action of NSAIDs, could play an important part in anti-rheumatic and anti-inflammatory actions. The anti-inflammatory effect of EOs may be due to the presence of oxygenated monoterpenes such as eucalyptol and linalool either singly or in combination [23,25].

### 2.3. In Vivo Anti-Inflammatory Activity Assay

#### 2.3.1. Carrageenan-Induced Paw Edema

Carrageenan-induced mice paw edema is often used to evaluate the anti-inflammatory effect of diverse natural bioactive compounds such as phytochemical extracts and EO. The anti-inflammatory activity of orally administered LSEO (2, 20, and 200 mg/kg) was determined using the same paw edema model. As shown in Table 4, in comparison with the NSAID indomethacin, LSEO exhibited a significantly high anti-inflammatory activity in a dose-dependent manner.

At 4 h after oral administration of LSEO, the degree of edema inhibition was similar for 20 mg/kg and 200 mg/kg (42.07% and 47.06%, respectively). This level of edema inhibition was comparable to the level observed using 25 mg/kg of the standard reference NSAID (44.5%).

Inflammatory illnesses are presently treated with steroidal and NSAIDs. Unfortunately, both of these widely prescribed treatment classes have important harmful side effects and fail in certain segments of the population [23]. Therefore, there is a need to develop and produce new treatments with novel mechanisms of action that do not generate significant side effects. It has been reported that a variety of EOs exhibit noticeable anti-inflammatory properties in numerous diverse models of inflammation. Investigations on the anti-inflammatory action of LSEO are limited. Only one research report suggested the aptitude of *Lavandula angustifolia* EO to reduce the carrageenan-induced paw edema in animals at doses of 200 mg/kg, even though the mode of action was not addressed in this publication [12]. The precise mechanism of the anti-inflammatory activity of the LSEO is unclear. Nevertheless, it has been reported that a number of constituents contribute to the incomplete reduction of the release of inflammation mediators. In recent years, numerous studies have reported oxygenated mono- and sesquiterpenes and their hydrocarbon derivatives as the main compounds of EOs, which have effective anti-inflammatory activity [5,26]. In our study, 1,8-cineole (eucalyptol) has been found to be the major compound in LSEO. It appears that 1,8-cineole can be partially linked with the observed pharmacological activity, but it is not apparent if the other oxygenated monoterpenes (fenchone, pinene) can also potentiate this effect. Our results are in agreement with those published for other EOs rich in 1,8-cineole that demonstrated a potent and strong anti-edematogenic effect [12,26,27]. They revealed that the EOs, which are rich in 1,8-cineole, showed analgesic and anti-inflammatory properties. Eucalyptol also exhibits an inhibitory effect in a number of tests of experimental inflammation in animals, using the carrageenan-induced paw edema test [26]. These activities may be linked with the aptitude of eucalyptol to suppress the arachidonic acid metabolism and cytokine production in human monocytes [27].

#### 2.3.2. Xylene-Induced Ear Edema

Because the LSEO demonstrated an anti-inflammatory effect in the carrageenan-induced paw edema assay, the anti-inflammatory activity of LSEO was further evaluated by the inhibition of xylene-induced ear edema in mice. Topical application of xylene on the left ears caused noticeable edema as indicated by the augmentation in the earplug weight of the left ear compared with the untreated right ear (Table 5).

In comparison with positive control (diclofenac topical gel), LSEO exhibited a powerful and effective anti-inflammatory activity in our experimental animal model. Diclofenac gel (Voltarène emulgène^®^ 1%) produced 15.17% inhibition of xylene-induced edema, and this effect was statistically different and lower (*p* < 0.05) than that observed with all tested doses of LSEO. Further, LSEO reduced the inflammatory response by 80.1% for 410 mg/kg, which is higher than the positive control (Betasone) (70.3%). To the best of our knowledge, this is the first study to prove that LSEO has a significant topical anti-inflammatory activity in vivo. Consistent with current data, our previous report [28] showed that topical application of EOs can limit the inflammatory symptoms of edema and neutrophil accumulation. In phytotherapy, dermal application of EOs in a full body massage or to limited parts of the body is greatly pleasing. Many EOs are used as curative ingredients for inflammatory indications with lesional neutrophil accumulation: aphthous stomatitis, rheumatoid arthritis, and lesional fungal or bacterial contagions [8,11,29].

#### 2.3.3. Mouse Ear Tissue Morphology

**Figure 2:**
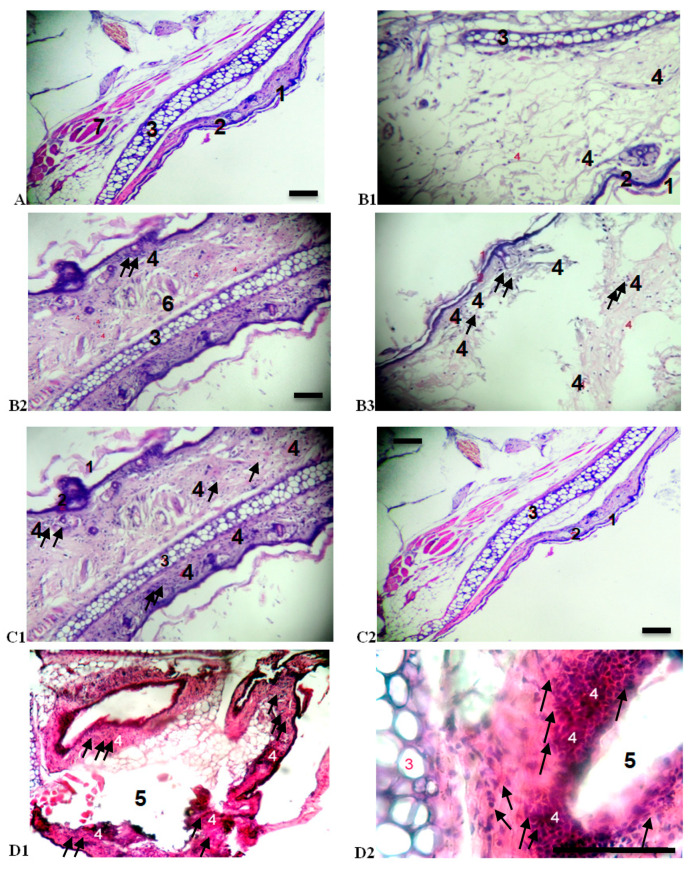
Sections of mice ear biopsies showing keratin, epidermal, dermal, muscle, and cartilage layers. Hematoxylin & Eosin stained sections were scored as mild (+), modest (++), and severe (+++) for edema and substantial inflammatory polymorphonuclear (PMN) cell infiltration in the dermis inflammation phase. (1) Keratin; (2) epidermal layer; (3) cartilage layer; (4) PMN; (5) edema; (6) muscle. (**A**) Right ear without treatment (×10). (**B**) *Lavandula stoechas* Essential Oil (LSEO) treatment with different doses (**B1**): LSEO 82 mg/kg (G × 10), (**B2**): LSEO 410 mg/kg (G × 10), (**B3**): LSEO 810 mg/kg (G × 10)) = edema (±); inflammatory cell infiltration (+), inflammation phase (±). (**C**) Positive control treatment (**C1**): Voltarène Emulgel (G × 10); (**C2**): betamethasone (G × 40) = edema (±); inflammatory cell infiltration (+), inflammation phase (±). (**D1**) Negative control (×10) = edema (++); inflammation phase (+++); inflammatory cell infiltration (+++) in epidermal and dermal layers, muscle, and cartilage. (**D2**) Negative control (×40). Scale − bar = 25 µm; black arrow indicates inflammatory polymorphonuclear cell infiltration.

We investigated H&E-stained ear sections from xylene-induced animals (Figure 2). Xylene is a highly irritating substance that stimulated an inflammatory response in the epidermis. Xylene application resulted in a noticeable increase in ear thickness with obvious confirmation of edema, epidermal hyperplasia, and inflammatory cell infiltration in the dermis with associated connective tissue disruption (Figure 2D1,D2).

By histological comparison, topical application of LSEO decreased ear thickness and associated pathological indicators to an extent comparable to the positive controls (sodium diclofenac and betamethasone gels) (Figure 2B,C). These findings directly demonstrate the properties of LSEO within the target tissue, providing additional confirmation that LSEO ameliorates xylene-induced contact dermatitis. Microscopic investigation showed the valuable anti-inflammatory activity of the topical application with LSEO. Compared to the control groups, edema was dramatically reduced by the previous topical treatment with LSEO (Figure 2A vs. Figure 2B). To the best of our knowledge, this is the first study to reveal that LSEO has a significant topical anti-inflammatory activity, which is confirmed by histology examination.

Current results are consistent with previous publications about other EOs using the carrageenan and xylene-induced edema methods [29,30,31]. In addition, the current results are in agreement with our previous research [32], in which histological analysis revealed that rose-scented geranium oil inhibited the skin inflammatory process in vivo. In conclusion, the results of our investigation support the traditional usage of LSEO as an anti-inflammatory agent, although there is a need for further investigations to better estimate its pharmaceutical potential and understand its mode of action.

### 2.4. Effects of LSEO on Cytotoxicity of Three Human Tumor Cell Lines

**Figure 3:**
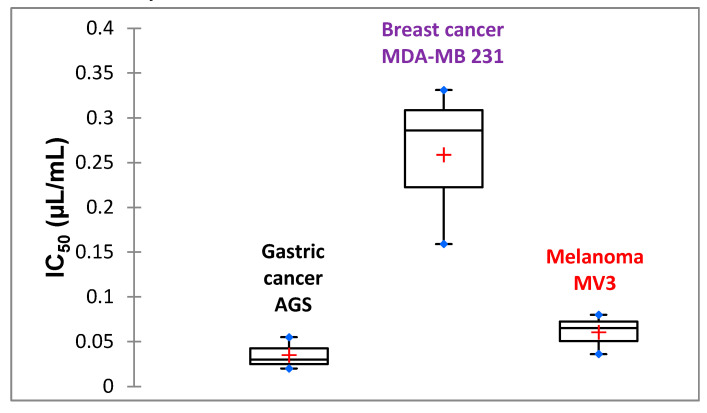
In vitro cytotoxic effect of LSEO against different cancer cell lines using MTT assay. IC_50_: Median inhibitory concentration. Experiments were performed three times in octuplets.

Because anti-proliferative screening models in vitro provide important preliminary data to help select compounds with potential antineoplastic properties for further study, the LSEO was tested in vitro for its potential human tumor cell growth inhibitory effect on human breast carcinoma MDA-MB-231, human gastric cancer AGS, and human melanoma MV3, using MTT assay. This is a non-radioactive, fast, and economical assay widely used to quantify cell viability and proliferation. As shown in Figure 3, LSEO had selective cytotoxicity on different tumor cells, and a potent anti-proliferative effect on AGS cells with IC_50_ value of 0.035 ± 0.018 µL/mL. This potent in vitro antitumor effect was also shown in MV3 and MDA-MB-231 cell assays, with IC_50_s of 0.06 ± 0.022 µL/mL and 0.259 ± 0.089 µL/mL, respectively. To the best of our knowledge, this is the first report on the anti-proliferative activity of LSEO.

**Figure 4:**
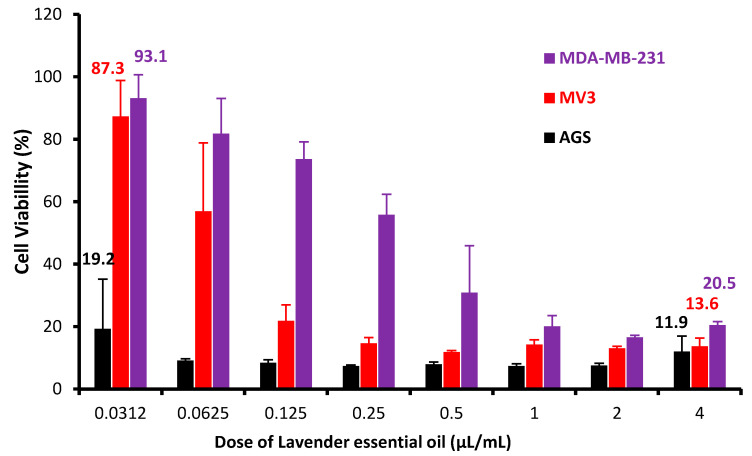
Anti-proliferative activity of LSEO after 24 h of exposure in the MTT assay. MDA-MB-231: human breast carcinoma cells; MV3: human melanoma; AGS: human gastric cancer. Experiments were performed three times in octuplets.

It is shown in Figure 4 that LSEO has an important dose-dependent cytotoxic effect against all cancer cell lines tested. At higher concentration (4 µL/mL *v*/*v*), LSEO was more cytotoxic against AGS cells (88.1% lysis) than MV3 cells ((86.4% lysis) and MDA-MB-231 cells (79.5% lysis). At low concentration (0.032 µL/mL *v*/*v*) of LSEO, this same order of sensitivity was also obtained. AGS was the most sensitive cancer cells (80.8%) at the low concentration.

A previous investigation of another lavender species (*L. angustifolia*) EO showed cytotoxicity to human skin cells in vitro at a concentration of 0.25% (*v*/*v*) [10]. As we did not evaluate the cytotoxic effect of all chemicals present in LSEO against the three cancer cell lines, it is not possible to identify which of these compounds are responsible for the observed results. It appears from this analysis that EOs containing a high amount of eucalyptol are more cytotoxic than the others [33]. These results are in agreement with those of other authors who reported that the eucalyptol (1,8-cineole) has an important antitumor effect against tumor cell lines like hormone-refractory prostate cancer [7] and drug-resistant human lung cancer [6]. Specific induction of apoptosis, not necrosis, was observed in human colon cancer cell lines HCT116 and RKO by 1,8-cineole. The treatment with 1,8-cineole was associated with inactivation of survivin and Akt and activation of p38. These molecules induced cleaved poly(ADP-ribose) polymerase (PARP) and caspase-3, finally causing apoptosis. In xeno-transplanted SCID mice, the 1,8-cineole group showed significantly inhibited tumor progression compared to a control group [33,34]. On the other hand, Tayarani-Najaran et al. [35] studied the cytotoxicity and the mechanisms of cell death induced by the EO of *Lavandula angustifolia*, and were compared with both normal (human fibroblast cells) and malignant cancerous human cells (HeLa Human cervix carcinoma and MCF-7 lung adenocarcinoma cell lines). They found that the IC_50_ for normal cells was higher (>500 µg/mL) than that reported with cancer cell lines (IC_50_ HeLa = 31.92 µg/mL; IC_50_ HeLa = 31.92 µg/mL); thus, confirming a higher sensitivity of tumor cells as compared to normal cells.

LSEO with an anti-proliferative activity shows also in vitro and in vivo anti-inflammatory properties. Even though there is a relationship between these two activities, the various mechanisms involved for each EO could explain why there is variability of these effects. A link between inflammation and cancer has long been suspected, but its molecular nature remains to be defined. Chronic inflammation may directly affect the cells that eventually become transformed as well as exert indirect effects on the tumor cell through surrounding cells [4,36,37]. In summary, the cytotoxic activity of LSEO might be due to the synergic effects of different terpenes in the oil, or perhaps there are some other active compounds responsible for the cytotoxic activity of the essential oil, which deserves attention in the future.

## 3. Materials and Methods

### 3.1. Material

#### 3.1.1. Extraction of *Lavandula stoechas* Essential Oil

*Lavandula stoechas* (Lamiaceae family) aerial parts were collected in 2016 in the region of Cherchell (Tipaza, Algeria). This area is located in the western region of Algiers and is situated at 36°34′3.014″ N and 12′14.376″ E in the central north of the country. LSEO was distilled from the leaves, stems, and flowers using alembic steam distillation. The process consists of passing water vapor at a high-pressure through an alembic (tank) filled with aromatic plants. The steam captures the volatile compounds that are confined in the secretory glands of the aromatic herb, which then pass through a cold-water frozen serpentine and condense into a liquid. Upon exit, phases of diverse densities are separated with the help of a “Florentine vase” and floral water and EO (also named “aromatic water”) are obtained.

#### 3.1.2. Solvents, Drugs and Chemicals

The following drugs and chemicals purchased from Sigma Chemical Co. (St. Louis, MO, USA) were used: dimethyl sulfoxide (DMSO), bovine serum albumin (BSA), sodium diclofenac, phosphate-buffered saline (PBS, 10 mM, pH 7.4), sterile saline solution (0.9% *w*/*v* NaCl), Alsever solution (2% dextrose, 0.8% sodium citrate, 0.5% citric acid, and 0.42% NaCl), 3-(4,5-dimethylthiazol-2yl)-2,5-diphenyl-tetrazolium bromide (MTT), xylene, Tween 80, acetone, and formaldehyde solutions. Voltarène emulgène^®^ 1% (diethylamine diclofenac, Novartis, Algeria), Betasone^®^ 0.05% (Betamethasone, Saidal Pharmaceuticals, Algiers, Algeria), and Indomet^®^ 25 mg (Indomethacin, Saidal Pharmaceuticals, Algiers, Algeria) were also used. Roswell Park Memorial Institute (RPMI)-1640 medium and other cell-culture reagents including fetal bovine serum (FBS), penicillin, streptomycin, and amphotericin B were obtained from Gibco Inc. (Grand Island, NY, USA).

#### 3.1.3. Animals

Swiss albino NMRI (The Naval Medical Research Institute, Institut Pasteur d’Algérie, Algiers, Algeria) mice of both sexes, weighing from 24–28 g and pathogen free, were obtained from animal breeding of the R&D Center of Saidal Pharmaceuticals and from the “Laboratoire National de Contrôle des Produits Pharmaceutiques” (Algiers, Algeria), respectively. The animals were left for 3 days at room conditions for acclimatization. A minimum of 5 animals were used in each group, and were kept at room temperature with a 12 h light/dark cycle. They were maintained on a standard pellet diet and water *ad libitum* throughout the experiment. The pellets for mice have been purchased from a commercial producer (National Livestock Food Office, Algiers, Algeria). Below is the composition of the pellets: Carbohydrates: 49.8%; Crude Protein: 23.5%; Crude Fat: 5.0%; Crude Fiber: 5.5%; Acid Insoluble Ash: 6.5%; Calcium: 1.1%; Phosphorus: 0.8%; Moisture: 12%; Vitamin A (UI/kg): 22,000; Vitamin D (UI/kg): 2000; Vitamin E (UI/kg): 100. All animal experiments have been conducted in accordance with directives approved by current institutional guidelines (Saidal Pharmaceuticals, Algiers, Algeria) for animal treatment (88-08/1988) and approved by the Council of the European Union (2010/63/EU) on the Protection of Animals Used for Scientific Purposes.

#### 3.1.4. Cancer Cell Lines

Three human cancer cell lines were used. MDA-MB-231 cells, which are estrogen receptor-negative human breast cancer; human melanoma MV3 cells derived from lymph node with a high metastatic potential; and human gastric adenocarcinoma (AGS) cells. Cell lines were obtained from the American Type Culture Collection (ATCC, Manassas, VA, USA). Cells were maintained as a monolayer culture in the RPMI-1640 nutrient medium and were grown at 37 °C in a humidified chamber with 5% CO_2_ as monolayer adherent cultures in 75 cm^2^ tissue culture flasks, in a medium supplemented with 10% FBS, 1% penicillin, and 1% streptomycin.

### 3.2. Methods

#### 3.2.1. Determination of the Chemical Composition of Essential Oil

Analysis and identification of the volatile compounds were performed using a Shimadzu GC-17A Gas Chromatograph coupled with a Shimadzu QP-5050A Mass Spectrometer detector (Shimadzu Corporation, Kyoto, Japan). The GC-MS system was equipped with a Tracsil Meta.X5 (95% dimethylpolysiloxane and 5% diphenylpolysiloxane) column (60 m × 0.25 mm, 0.25 μm film thickness). Analyses were carried out using helium as the carrier gas at a column flow rate of 0.3 mL/min and a total flow of 3.9 mL/min in a split ratio of 1:200 and the following program: (a) 80 °C for 0 min; (b) increase of 3 °C/min from 80 °C to 210 °C and hold for 1 min; (c) increase of 25 °C/min from 210 °C to 300 °C and hold for 3 min. The temperatures of the injector and detector were 230 °C and 300 °C, respectively. All compounds were identified using two different analytical methods: (1) comparison of experimental retention indexes (RI) with those of the literature; (2) mass spectra (authentic chemicals and National Institute of Standards and Technology (NIST05) spectral library collection). Only fully identified compounds are reported in this study.

#### 3.2.2. In Vitro and In Vivo Anti-Inflammatory Activities

##### Anti-Inflammatory Test Using Erythrocyte System Cellular Model In Vitro

Preparation of blood samples for membrane stabilization assays: the human red blood cells (HRBC) membrane stabilization method has been used as a method to study the in vitro anti-inflammatory activity [23]. Human venous blood samples were freshly collected under informed consent from a healthy human volunteer, and put into test tubes containing anticoagulant (EDTA-Na_2_ 10%) and mixed with equal volume of Alsever solution. Blood samples were centrifuged at 2500 rpm for 5 min and the supernatant was removed. The cell suspension was washed with sterile saline solution and centrifuged at 2500 rpm for 5 min. This was repeated three times until the supernatant was clear and colorless and the packed cell volume was measured. The cellular component was reconstituted to a 40% suspension (*v*/*v*) with PBS (10 mM, pH 7.4) and was used in the assays.

Hypotonicity-induced hemolysis: Different concentrations of LSEO (6–0.4 µL/mL) were prepared using distilled water, and to each concentration 1 mL of PBS, 2 mL hyposaline and 0.5 mL of HRBC suspension were added. It was incubated at 37 °C for 30 min and centrifuged at 3000 rpm for 20 min. The hemoglobin content of the supernatant solution was estimated spectrophotometrically at 560 nm. Sodium diclofenac as a non-steroidal anti-inflammatory drug (NSAID) was used as a reference standard and a control was prepared by omitting the LSEO. The percentage inhibition of hemolysis or membrane stabilization was calculated according to the method described by Parvin et al. [21].

$$\mathrm{Inhibition}\;\mathrm{of}\;\mathrm{hemolysis}\;\left(\%\right)=\left(\frac{A1-A2\;\mathrm{sample}}{A1}\right)\times 100$$

where: A1 = optical density of hypotonic-buffered saline solution alone and A2 = optical density of test sample in hypotonic solution.

##### Anti-Inflammatory Activity Using the Inhibition of Denaturation of Albumin In Vitro

The capability of LSEO to inhibit the denaturation of BSA was investigated by a method as reported by Rahman et al. [22]. Typically, different concentrations (6–0.4 µL/mL) of LSEO were prepared and the volumes were adjusted to 2.5 mL with 0.85% NaCl. Then 0.5 mL of BSA (5 mg/mL) was added. The mixture was incubated at 37 °C for 20 min and further incubated at 55 °C for 30 min. The tubes were cooled and 2.5 mL of 0.5 M PBS (pH 6.3) was added. The turbidity was measured spectrophotometrically at 560 nm. The assay was carried out in triplicates and the standard (sodium diclofenac) was used in place of the LSEO. Percentage inhibition of BSA denaturation was evaluated as follow:

$$\%\;\mathrm{Inhibition}\;=\left(\frac{\mathrm{Abs}\;\mathrm{Control}\;-\mathrm{Abs}\;\mathrm{Test}}{\mathrm{Abs}\;\mathrm{Control}}\right)\times 100$$

##### In Vivo Anti-Inflammatory Assay Using Carrageenan-Induced Paw Edema

This technique is based on a report by Bouhlali et al. [23]. Carrageenan is known to result in at least neutrophil-linked edematous inflammation. LSEO was diluted in 0.5% Tween 80 and administered 30 min prior to carrageenan injection. The control group received an equivalent volume of the vehicle (0.5% Tween 80 in 0.9% NaCl solution). LSEO at doses of 2, 20, or 200 mg/kg and vehicle were administrated *per os* (*p.o.*) 30 min before injecting the carrageenan. Paw edema was induced with a single 0.1 mL sub-plantar injection of carrageenan (0.1 mL) into the left hind paw of conscious mice. Indomethacin (25 mg/kg, *p.o.*) was used as positive control (NSAID). The mice were sacrificed 4 h later. The difference in weight between right untreated and left treated hind paws was calculated and results are expressed as the increase in paw weight (mg). The percentage inhibition of the inflammatory response was calculated for each mouse by comparison to the negative control, and calculated using the following formula:

$$\%\;\mathrm{Inhibition}\;\mathrm{of}\;\mathrm{edema}\;=\left(1-\frac{\Delta \mathrm{Pt}}{\Delta \mathrm{Pc}}\right)\times 100$$

where ΔPt is the difference in paw weight in the drug-treated group, and ΔPc is the difference in paw weight in the control group.

##### In Vivo Anti-Inflammatory Activity Using Xylene-Induced Ear Edema

Topical anti-inflammatory activity was assessed as inhibition of xylene-induced ear edema in mice [23]. Male Swiss mice were divided into groups of 5 mice each. Thirty min after the dermal application of LSEO, 10 µL acetone solution containing 10% xylene was carefully applied to the anterior and posterior surfaces of the left ear. The right ear remained untreated. Vehicle (sweet almond oil), doses of LSEO diluted in almond oil (82, 410, and 820 mg/kg), and positive controls (Voltaren^®^ Emulgel 1% and Betasone^®^ 0.05%) were applied topically to the left ear about 30 min before the xylene application. At the maximum of the edematous response (4 h later), mice were sacrificed and a plug (5 mm in diameter) was removed from both treated (left) and untreated (right) ears. The edema response was calculated as the weight difference between the two plugs. LSEO anti-inflammatory potential was expressed as percentage of the edema weight reduction in treated mice in comparison to the control group, and calculated using the following formula:

$$\%\;\mathrm{Inhibition}\;\mathrm{of}\;\mathrm{edema}\;=\left(1-\frac{\Delta \mathrm{Wt}}{\Delta \mathrm{Wc}}\right)\times 100$$

where ΔWt is the change in weight of ear tissue in the treated mice, and ΔWc is the change in weight of ear tissue in the control mice.

##### Morphological Analysis of Ear Tissue

The resulting inflammatory response was checked and monitored by measurement of edema formation and by microscopic observation. For morphological examination of cutaneous inflammation, biopsies from control and treated ears of animals were collected at the end of the experiment. Samples were fixed using 10% neutral buffered formalin, routinely processed, and sectioned at 6 µm using a microtome (Leica RM 2125RT, Nussloch, Germany). Sections were stained with Hematoxylin and Eosin (H&E) and the tissues were observed with a light microscope (Olympus CX41, Olympus, Tokyo, Japan) and graded as mild (+), moderate (++), and severe (+++) for inflammation phase. Infiltration and polymorphonuclear (PMN) cells’ accumulations were also assessed [29].

#### 3.2.3. Cytotoxic Activity Using MTT Test

MDA-MB-231, AGS, and MV3 cells were grown in RPMI-1640 medium supplemented with 10% FBS, penicillin (100 U/mL) and streptomycin (100 µg/mL). Cells were maintained at 37 °C in a humidified incubator with 5% CO_2_ and regularly examined using an inverted microscope. The medium was replaced every two days and cells were sub-cultured at 70%–80% confluence. Cells in 96-well plates (100 µL/well) were exposed to different concentrations of LSEO (4 to 0.0312 µL/mL) in DMSO/RPMI (0.1% *v*/*v*) at 37 °C and 5% CO_2_ for 24 h. Final DMSO concentration did not affect cell viability. The MTT colorimetric assay was used to evaluate the cytotoxic effect of LSEO [5]. MDA-MB-231, AGS, and MV3 cells were placed into 96-well culture plates (10 × 10^3^ cells per well) and incubated for 24 h. Then, 100 µL of culture medium containing the specified concentration of the LSEO was added to each well. After exposure to serial concentrations of LSEO for 24 h at 37 °C and 5% CO_2_, 100 µL of medium were carefully aspirated from each well and replaced by 100 µL of MTT solution (5 mg/mL in PBS). After addition of 100 µL PBS containing 0.5 mg/mL MTT, cells were incubated at 37 °C for 4 h. Formed formazan crystals were dissolved in 50 µL DMSO. Absorbances in the control and LSEO-treated wells were measured at 490 nm with an ELISA reader (BioTek, Winooski, VT, USA). Growth inhibition was calculated as follows:

$$\%\;\mathrm{cell}\;\mathrm{viability}=\left(\frac{\mathrm{Abs}\;\mathrm{Test}}{\mathrm{Abs}\;\mathrm{Control}}\right)\times 100$$

Concentration median inhibitory concentration (IC_50_) (µL/mL) was defined as the concentration of LSEO producing 50% inhibition of cell survival. It was determined from the cell survival diagrams.

#### 3.2.4. Statistical Analysis

Mean values of treated groups were compared with those of a control group and analyzed using statistical methods. Data are reported as mean ± standard deviation (SD). Comparison between different groups was conducted with one-way analysis of variance (ANOVA) followed by Tukey’s post hoc multiple comparison test. Differences with *p* < 0.05 between experimental groups were considered statistically significant. IC_50_ (median inhibitory concentration) was calculated from the dose response curve obtained by plotting percentage inhibition versus concentrations. Statistical data analysis was performed using XLStat 2014 software (Pro statistical software, Addinsoft, Paris, France).

## 4. Conclusions

In conclusion, we describe for the first time the anti-inflammatory and anticancer effects of LSEO and its chemical composition. Our results show that LSEO has important in vitro and in vivo anti-inflammatory and cytotoxic effects against melanoma, breast cancer, and gastric cancer cells. These data may serve as valuable research references for clinical research of medicines for treatment of inflammation and cancer in the future and also a tool promoting the use of therapeutic benefits of EOs. Considering that drugs currently available for the treatment of inflammatory and cancer conditions show undesirable side effects, the present results may have clinical relevance and open new possibilities for the development of novel anti-inflammatory and anticancer drugs. Further studies to elucidate the mechanisms of action, and the possible compounds involved in these activities, will be undertaken.

## Figures and Tables

**Table 1:** Chemical composition of the volatile oil extracted from *Lavandula stoechas* using a steam distillation method.

Retention Time (min)	Name	%
8.874	α-Pinene	4.75
9.155	Camphene	0.35
9.808	β-Pinene	13.83
11.031	1,8-Cineole	61.36
11.640	*cis*-Linalool oxide	0.92
11.887	*trans*-Linalool oxide	1.39
12.114	Linalool	1.63
12.475	α-Campholenal	0.44
12.734	Pinocarveol	2.12
12.778	Camphor	0.63
13.067	Pinocarvone	2.04
13.181	α-Terpineol	3.15
13.288	Terpineol-4	0.96
13.453	Cryptone	0.59
13.532	α-Terpineol	1.14
13.598	Myrtenal	2.36
13.781	Verbenone	0.29
14.261	Carvone	0.21
17.442	Aromadendrene	0.98
17.749	δ-Elemene	0.66
17.863	*cis*-Calamenene	0.20
	Oxygenated Monoterpenes	79.23
	Monoterene Hydrocarbons	18.93
	Sesquiterpene Hydrocarbons	1.84

**Table 2:** Effect of LSEO on stabilization of HRBC membrane in vitro.

Treatment	Concentration	Absorbance (560 nm)	% Inhibition of Hemolysis	IC_50_^#^
**Control (PBS)**		0.568	-	-
LSEO (µL/mL)	6	0.370	34.917 ± 1.939 ^D^	6.214 ± 0.776 ^B^
	3	0.145	74.471 ± 0.465 ^C^	
	1.5	0.064	88.791 ± 0.101 ^B^	
	0.8	0.042	92.605 ± 0.000 ^A^	
	0.4	0.042	92.605 ± 0.465 ^A^	
Sodium diclofenac (mg/mL)	30	0.46	19.014 ± 12.707 ^F^	1.198 ± 0.735 ^A^
	3	0.411	27.552 ± 3.354 ^E^	
	0.3	0.045	92.165 ± 0.419 ^A^	
	0.03	0.041	92.693 ± 0.227 ^A^	
	0.003	0.04	92.913 ± 0.221 ^A^	

Each value represents the mean ± SD. LSEO: *Lavandula stoechas* Essential Oil. IC_50_: Median Inhibitory Concentration; HRBC: Human Red Blood Cells; PBS: phosphate-buffered saline. ^#^ Means within the same column followed by the same letter are not significantly different (*p* > 0.05) according to ANOVA analysis followed by Tukey’s post hoc multiple comparison tests.

**Table 3:** Effect of LSEO on heat-induced protein denaturation.

Treatment	Concentration	Absorbance (560 nm)	% Inhibition of BSA	IC_50_^#^
**Control (PBS)**		1.288	-	-
LSEO (µL/mL)	6	0.067	62.569 ± 0.967 ^E^	2.447 ± 0.873 ^A^
	3	0.061	65.735 ± 0.853 ^D^	
	1.5	0.054	69.832 ± 0.558 ^C^	
	0.8	0.048	73.184 ± 0.558 ^B^	
	0.4	0.049	72.625 ± 2.560 ^B^	
Sodium diclofenac (mg/mL)	10	0.165	7.960 ± 7.741 ^F^	8.260 ± 0.943 ^B^
	1	0.04	77.932 ± 0.721 ^A^	
	0.1	0.044	75.279 ± 2.555 ^A^	
	0.01	0.043	76.117 ± 0.534 ^A^	
	0.001	0.045	74.720 ± 0.279 ^AB^	

Each value represents the mean ± SD. LSEO: *Lavandula stoechas* Essential Oil. IC_50_: Median Inhibitory Concentration; BSA: Bovine Serum Albumin. ^#^ Means within the same column followed by the same letter are not significantly different (*p* > 0.05) according to ANOVA analysis followed by Tukey’s post hoc multiple comparison tests.

**Table 4:** In vivo anti-inflammatory effect of LSEO using carrageenan induced-paw edema.

Treatment (Dose μg/kg)	Weight (mean, mg) ± SD			% Inhibition of Edema
	Left Hind Paw	Right Hind Paw	Edema Weight ^#^	
LSEO (200)	126.10 ± 8.00	110.12 ± 7.12	15.975 ± 7.31 ^A^	47.0588
LSEO (20)	138.48 ± 10.1	121.00 ± 6.72	17.480 ± 8.71 ^A^	42.0712
LSEO (2)	146.08 ± 8.61	122.35 ± 6.00	23.733 ± 9.22 ^A,B^	21.3476
Positive control (Indomethacin)	161.25 ± 6.12	144.50 ± 6.18	16.750 ± 7.50 ^A^	44.4904
Negative control	154.30 ± 7.65	124.12 ± 10.0	30.175 ± 13.41 ^B^	/

Groups of animals (*n* = 5 mice per group) were pretreated with vehicle, Indomethacin (25 mg/kg, *p.o*.) or LSEO at doses of 2, 20, and 200 mg/kg *per os* (*p.o.*) 30 min before carrageenan-induced paw edema. LSEO: *Lavandula stoechas* Essential Oil. ^#^ Means within the same column followed by the same capital letter are not significantly different (*p* > 0.05) according to ANOVA analysis followed by Tukey’s post hoc multiple comparison test.

**Table 5:** *Lavandula stoechas* aromatic oil prevents xylene-induced ear edema in mice.

Treatment (Dose mg/kg)	Weight (mean, mg) ± SD			% Inhibition of Edema
	Left Ear	Right Ear	Edema Weight ^#^	
LSEO (820)	22.97 ± 4.13	16.52 ± 0.73	6.45 ± 2.80 ^B^	25.8620
LSEO (410)	16.37 ± 1.92	14.65 ± 2.22	1.72 ± 1.20 ^A^	80.1724
LSEO (82)	22.72 ± 5.16	18.50 ± 1.99	4.22 ± 2.88 ^B^	51.4367
Betasone^®^ 0.5%	18.08 ± 2.79	15.50 ± 1.50	2.58 ± 1.49 ^A,B^	70.3448
Voltarene Emulgel^®^ 1%	20.70 ± 4.94	13.32 ± 1.35	7.38 ± 3.17 ^B^	15.1724
Negative control (Vehicle)	28.10 ± 6.12	19.40 ± 4.13	8.70 ± 3.55 ^C^	/

Data are presented as Mean (mg) ± Standard Deviation (SD) (*n* = 5 mice per group). LSEO: *Lavandula stoechas* Essential Oil. ^#^ Means within the same column followed by the same capital letter are not significantly different (*p* > 0.05) according to ANOVA one way analysis followed by Tukey’s post hoc multiple comparison test.

## Abbreviations

ANOVA	analysis of variance
BSA	bovine serum albumin
DMSO	dimethyl sulfoxide
EO	essential oil
FBS	fetal bovine serum
GC-MS	gas chromatography-mass spectrometry
H&E	hematoxylin-eosin
HRBC	human red blood cells
IC_50_	median inhibitory concentration
LE	left ear
LHP	left hind paw
LNCPP	Laboratoire National de Contrôle des Produits Pharmaceutiques
LSEO	*Lavandula stoechas* essential oil
MTT	3-(4,5-dimethylthiazol-2yl)-2,5-diphenyl-tetrazolium bromide
NIST	National Institute of Standards and Technology
NMRI	Naval Medical Research Institute
NSAID	non-steroidal anti-inflammatory drugs
PBS	phosphate-buffered saline
*p.o.*	*per os*
PARP	poly(ADP-ribose) polymerase
PMN	polymorphonuclear cells
R&D	Research and Development Center
RE	right ear
RHP	right hind paw
RI	retention index
rpm	rotation per minute
RPMI	Roswell Park Memorial Institute
RT	retention times
